# On the Use of Strychnine in Epilepsy

**Published:** 1868-05

**Authors:** Walter Tyrrell

**Affiliations:** Malvern


### ON THE USE OF STRYCHNINE IN EPILEPSY.

By WALTER TYRRELL, Esq., Malvern.

[Mr. Tyrrell published a paper on this subject in May, 1867.]

I stated at the end of my former paper, my belief that "in strychnine we possess a drug which will always control the excitability of the medulla oblongata, and restrain the attacks of convulsion." This opinion will, I think, be found to be remarkably strengthened by a perusal of the following cases. One most important fact is to be gathered from them, *viz.*, that large doses of the drug must be given to produce the favorable results. In some of the cases the doses may appear formidable, but I feel confident that with care and watchfulness no ill effects need follow their administration to the epileptic. In such cases the system appears to lose its susceptibility; and the drug, even in large doses, produces none of the ordinary signs of disagreement. In no case have I seen it produce any mischievous excitement or irritation; and I may state that in one very severe case, still under treatment, I have carried the dose as high as one-fifth of a grain, taken twice daily, and this continued for nearly three weeks together, not only without its producing the slightest sign of irritation, but, on the contrary, the most marked diminution in the frequency and violence of the attacks. The following case, although the attacks had but recently come on, is interesting, as showing how rapidly the beneficial effect of strychnine is often gained, no attacks having supervened after two doses (each of one-twelfth of a grain) had been taken.

H. R., aged 29, has been of late years much exposed to heat in China, Singapore, and Japan; had congestion of the liver in March, 1865; was invalided home in June, 1865; since which time he has been living at home, under treatment for enlargement of the liver, using iodide of potassium and iodine ointment locally. On May 22, of this year—a very cold, snowy day—he imprudently stayed out all day fishing, and at dinner that evening was seized with a violent epileptic fit, accompanied with great convulsion; this was followed by other attacks at the following intervals: May 25, three fits, at intervals of one hour and a-half; May 30, a fit in the evening; June 1, two fits, with six hours' interval; June 2, one fit in the evening. On June 5, he arrived in Malvern, and I prescribed for him one-twelfth of a grain of strychnia twice daily, allowing him to continue his potash in rather increased quantity. On the morning of the 6th, he had three fits, during the first of which I was present; they were very convulsive, and produced an extremely prostrating effect on his mind—so much so, that even after the ordinary stupor had passed off, he was unable to answer the simplest question without consideration and great hesitation. It is needless to give a daily report of this case. I increased the dose of the strychnine to one-eighth of a grain; he had no further attacks; and his return to health, both bodily and mental, although gradual, was most perfect. He is now at the seaside, and may be considered, to all intents and purposes, convalescent. In this case, it was curious to observe how the inclination to an attack (which occurred several times during the early treatment of the case) yielded at once to a slight increase in the strength of the dose. I may say that in this case I found the use of ice to the nape very useful, insuring quiet sleep, and also allaying a frequent tendency to irritability.

In the following case, where the attacks were dependent on menstrual irregularity, the utility of combining the strychnine with remedies directed to the removal of the exciting cause will be apparent.

L. A., aged 17, a not unhealthy looking girl, has never menstruated properly; has been subject to epilepsy for four years, the interval never having been longer than one week; the attacks vary in intensity, a slight one being sooner followed by others. In this case, I commenced with one-sixteenth of a grain of strychnia twice daily, gradually carried up to the tenth, at the same time giving her aloes and myrrh, and assafœtida in pills twice daily. In this case, a perfect immunity from attacks commenced with the treatment, and has continued up to the present day, a period of nearly three months. Although the menstrual irregularity has not entirely ceased, it is very much ameliorated. I used, also, in this case, the cold effusion to the nape, coupling it at times with the application of warmth to the feet. This case, although not severe, is a type of a very prevalent form of the disorder, and shows how amenable such cases are to treatment. In another case, somewhat similar, which is still under treatment, I have the greatest benefit from the use of the bromide of potassium in combination with strychnia.

E. H., aged 14, a fair girl, partially paralyzed on the left side. When two years old had what was called brain fever, during which she was insensible for a length of time; recovered, but had a return about two years ago. Since the first attack she has been subject to continued attacks of *petit mal.*, sometimes five and six in the day. She turns slightly to the right; is slightly convulsed; sometimes is partially conscious during them, and tries to talk; sometimes she bites her tongue; her manner is silly, being fond of repeating lines of poetry, for which her memory is good. She has slight tenderness on pressure over the upper cervical vertebræ, and on percussing the atlas with the finger points she complains of pain at the epigastrium. The attacks sometimes come on during sleep. I will give here an extract from the diary kept by the parents. The patient came under my care in May, and I prescribed for her: ℞. Tinct. nucis vomicæ ʒiij, syr. aurantii ℥j. M., cap. ʒj bis in die ex aqua.

The following is a diary from May 29 up to the stoppage of the attacks:

May 29. Four fits in the day; two in the night.

30th. No fits in the day, but eight in the night, two of them being severe.

31st. One fit in the morning; eight again at night, but less severe.

June 1. No fit in the day; four at night.

2d. No fit in the day; five at night.

3d. No fit in the day; four at night.

4th. No fit in the day; three at night.

5th. No fit in the day; three slight ones at night.

6th. No fit in the day, and if any at night very slight.

7th. No fit in the day; only one observed at night.

8th. No fits day or night.

9th. No fits day or night.

10th. No fit.

11th. No fit.

12th. No fit.

And so on. Since this date she has continued almost entirely free from attacks, but few having occurred, and those of an altered and much slighter character, which yield readily to a slightly increased dose of the strychnine.

Although the following case is still under treatment, yet I think a slight sketch of it cannot fail to be interesting as exhibiting the effects of strychnine in very severe convulsive epilepsy, and as also showing what large doses of the drug may be given with impunity. This patient who has suffered for some years, is one of the severest cases of the disorder I have ever seen. I commenced treating him on the 15th June last, and as the case is still under treatment, I will merely give a comparative table of the number of his attacks during May of the present year and July. During the former month, he was under no treatment of any kind. During July, he was taking strychnine in the doses appended to the table. The attacks, which occurred almost exclusively at night, were most violently convulsive. They were much influenced by atmospheric changes, heavy thundery weather invariably increasing both their number and severity. Thus July would, under ordinary circumstances, be his most unfavorable month. In addition to the strychnine, during part of the month, he was using cold affusion to the nape and ice to the occiput during the night.

*No Treatment.*

| | | | |
|---|---|---|---|
| May 1, 1867, | 2 fits | May 17, 1867, | 1 fit |
| " 2, " | 1 | " 18, " | 0 |
| " 3, " | 2 | " 19, " | 0 |

| | | | |
|---|---|---|---|
| " 4, " ........... | 3 | " 20, " ........... | 0 |
| " 5, " ........... | 2 | " 21, " ........... | 1 |
| " 6, " ........... | 3 | " 22, " ........... | 0 |
| " 7, " ........... | 2 | " 23, " ........... | 2 |
| " 8, " ........... | 2 | " 24, " ........... | 2 |
| " 9, " ........... | 1 | " 25, " ........... | 3 |
| " 10, " ........... | 0 | " 26, " ........... | 3 |
| " 11, " ........... | 2 | " 27, " ........... | 2 |
| " 12, " ........... | 2 | " 28, " ........... | 1 |
| " 13, " ........... | 4 | " 29, " ........... | 2 |
| " 14, " ........... | 3 | " 30, " ........... | 0 |
| " 15, " ........... | 3 | " 31, " ........... | 0 |
| " 16, " ........... | 3 | | |

*Under Strychnine.*

| | Fits. | | | Fits. | |
|---|---|---|---|---|---|
| July 1, 1867, | 0 | $\frac{1}{8}$ gr. | July 16, 1867, | 3 | |
| " 2, " | 1 | very slight, | " 17, " | 0 | $\frac{1}{5}$ gr. |
| " 3, " | 1 | very slight, no con. | " 18, " | 0 | |
| | | | " 19, " | 0 | |
| " 4, " | 0 | | " 20, " | 0 | |
| " 5, " | 0 | | " 21, " | 0 | |
| " 6, " | 0 | | " 22, " | 0 | |
| " 7, " | 1 | | " 23, " | 0 | |
| " 8, " | 2 | | " 24, " | 0 | |
| " 9, " | 0 | $\frac{1}{6}$ gr. | " 25, " | 0 | |
| " 10, " | 0 | | " 26, " | 0 | |
| " 11, " | 0 | | " 27, " | 1 | |
| " 12, " | 0 | | " 28, " | 1 | |
| " 13, " | 0 | | " 29, " | 0 | |
| " 14, " | 1 | $\frac{1}{12}$ gr. | " 30, " | 0 | |
| " 15, " | 0 | | " 31, " | 0 | |

On July 14, owing to a misunderstanding, he only had $\frac{1}{12}$ of a grain instead of $\frac{1}{6}$. It will be seen that four fits followed in rapid succession. I think this table shows the power which strychnine possesses in restraining the epileptic attacks. I may add that, although so remarkably lessened in number, they were not at all increased in severity, but, on the contrary, were less convulsive. The above table gives the following results:

| MAY. | | JULY. | |
|---|---|---|---|
| No. of attacks. | Nights free. | No. of attacks. | Nights free. |
| 51 | 7 | 11 | 23 |

It will be observed that during the latter half of the month, the dose of strychnine was as high as $\frac{1}{5}$ of a grain, taken twice daily, and this without its producing the slightest sign of excitement or irritation. In combination with the strychnine, the patient is taking the infusion of digitalis.

In conclusion, I would reiterate the summing up of my last paper: "That in strychnine we possess a drug which will *always* control the excitability of the medulla oblongata and prevent convulsions, but that to cure the disease we must also remove the exciting cause.—*Medical Times & Gazette, Aug. 24, 1867, p. 201.—Braithwaite's Retrospect.*

